# Is Stryd critical power a meaningful parameter for runners?

**DOI:** 10.5114/biolsport.2023.118025

**Published:** 2022-09-15

**Authors:** Chey G. Dearing, Carl D. Paton

**Affiliations:** 1 The Eastern Institute of Technology, School of Health and Sport Science and School of Nursing, Napier, New Zealand; 2 The Eastern Institute of Technology, School of Health and Sport Science, Napier, New Zealand

**Keywords:** Endurance training, Athletic performance, Running, Wearable technology, Cardiorespiratory fitness, Anaerobic threshold

## Abstract

Stryd is a foot pod that reliably estimates running power. Our objectives were to examine the efficacy of the website-generated Stryd critical power (CP_STRYD_) as a meaningful parameter for runners. 20 runners performed their regular training while wearing Stryd for a minimum of 6 weeks to generate CP_STRYD_. Runners completed laboratory graded exercise testing, and outdoor 1500 m and 5000 m time trails. CP_STRYD_ was most similar to the second ventilatory threshold (VT2) or the onset of blood lactate accumulation (OBLA) and is highly predictive of running performance. Stryd ground contact time (GCT) was a predictor of performance when comparing runners at the same submaximal treadmill speed. CP_STRYD_ generated from outdoor running is equivalent to that calculated using an established CP model. However, variance between different methods of CP estimation must be a consideration for runners and coaches. Stryd offers meaningful data for runners including a realistic estimate of CP.

## INTRODUCTION

Bicycle mounted power meters have revolutionised cycling racing and training by allowing real-time measures of a rider’s power output. These bicycle power meters measure the direct force being applied to a component such as the pedal axle or crank arm using strain gauges. However, currently it is not possible to measure the power output of a runner using similar strain gauges. As an alternative, several manufacturers have developed devices to estimate running power using motion sensor units combined with complex power estimation algorithms [1]. One such running device is the Stryd power meter (Stryd Inc. Boulder CO, USA). The Stryd is a 9-gram foot pod that attaches to a running shoe and estimates power, pace, distance, vertical oscillation, cadence, leg spring stiffness and ground contact time (GCT). Stryd also estimates form power, and form power ratio which are estimates of running economy.

Stryd has been evaluated during treadmill running [2–5], track running [3] trail running [6] and walking [6, 7]. Stryd provides reliable measures for power [2–4, 7], though a minimum sampling time of 10 seconds is required at a constant speed [8]. Stryd also appears to offer reliable measurements of GCT [5, 9, 10]. Stryd compared with the OptoGait system exhibited greater precision for GCT at every speed examined [10]. Stryd was found to be valid for GCT during running when compared with RunScribe [5] though may underestimate GCT when compared with high-speed video analysis [5]. In contrast, when Stryd was compared at walking paces with the gold standard Optojump Next [7], Stryd overestimated GCT. While Stryd offers high reliability, bias between different sensor brands including Stryd are frequently noted.

Stryd offers its own website-based calculation of Critical power (CP). CP is a well-described endurance exercise concept: For any individual, time-to-exhaustion is highly predictable and directly related to the power output [11]. CP represents a boundary between steady-state and non-steady state exercise intensity [12] and may be related to skeletal muscle capillarity and proportions of slow twitch muscle fibre type in endurance-trained individuals [13, 14]. Essentially, CP is the highest intensity that can be sustained without accumulating significant fatigue from anaerobic metabolism. However, there is much controversy concerning CP as a threshold measure. CP values depend upon the trial protocols used and the mathematical models employed [15]. There is no accepted reference method for CP determination, however linear CP models that include trials of longer duration (> 10 minutes) are recommended [15]. A large range of exercise laboratory derived thresholds have been suggested to be comparable to CP intensity. These include ventilatory thresholds (VT1 and VT2), the lactate threshold (LT), the maximal metabolic steady-state (MLSS), the onset of blood lactate accumulation (OBLA) and even much higher intensities above VO_2peak_ (see [11, 16, 17] for reviews). Recent evidence suggest differences between CP and such thresholds may be due to discordance between the methods of determination [18]. Other authors have claimed that CP may differ significantly from other thresholds [16] and may be viewed controversially [19] as an independent physiological threshold itself. It has also been suggested that CP is simply a mathematical artefact [17].

To our knowledge, no study has examined the efficacy of the website generated CP_STRYD_ as a meaningful parameter for runners. It is currently unknown what relationship CP_STRYD_ has with other validated physiological thresholds including CP nor its ability to predict running performance. We sought to provide evidence to accept or reject CP_STRYD_ as a method to estimate CP or other thresholds in running. Our primary aim was to compare CP_STRYD_ with a CP calculated using an established linear power-time^−1^ model. Specifically, we sought to establish if the two CP models were equivalent. Secondary aims were to establish which lactate or gas exchange threshold is most equivalent to CP_STRYD_ and to compare CP_STRYD_ with standard laboratory performance tests as predictors of running performance. If CP_STRYD_ is equivalent to an established CP linear power-time^−1^ model and allows training at individual physiological thresholds and / or prediction of running performance, it will provide evidence to accept CP_STRYD_ as a meaningful parameter for runners.

## MATERIALS AND METHODS

### Participants

Twenty participants provided written informed consent prior to participation in this study that was approved by the Institutional Ethics Committee. The cohort consisted of males (n = 16), and females (n = 4) and included internationally competitive runners (n = 2), national class runers (n = 3) and competive recreational runners (n = 15). Inclusion criteria of participants were regular running training for the previous 3 months at a minimum frequency of 3 days per week, and currently participating in competitive running events and free from injury and illness.

### Laboratory testing with Stryd

All participants were familiar with treadmill running. Participants completed laboratory running tests in an environmentally controlled laboratory to minimise thermal strain [20] (temperature 15 ± 1°C: relative humidity 36 ± 4%) while wearing Stryd. Participants’ heart rate was recorded during all testing with Polar OH1 (Polar Electro Oly, Kempele, Finland). Participants completed all testing using a motorised treadmill (Cosmos pulsar 3p, HP Cosmos Sports and Medical GMBH, Nussdorf Traunstein, Germany). Prior to laboratory testing, participants were instructed to prepare as if it was a competition, to avoid strenuous physical activity and performance altering supplements (e.g. caffeine).

Participants first completed a graded exercise test (GET) to volitional exhaustion. Participants supplied their recent (within 4 months) race times from flat course races. Starting pace was 70% of actual or predicted 10000 m pace as recommended [21]. Mean starting speed was 9.8 ± 1.8 km · h^−1^. Stage increments were individualized to allow each participant to complete a minimum of five stages. 12.0 km · h^−1^ was one velocity that all runners maintained for one stage during testing. For this reason, the oxygen cost of running (VO_2_ L min^−1^) and Stryd economy estimates (form power and form power ratio) were examined for relationships at 12.0 km · h^−1^.

The gradient was fixed at 1% in order to simulate on-road conditions [22]. Prior to the first stage all participants completed a 10-min warm-up at a self-selected submaximal intensity. Each stage lasted four minutes with one-minute passive rest between stages to allow for the collection of capillary blood samples. A 5 ul blood sample was collected via finger prick and was measured for lactate using a portable analyser (Lactate Pro 2, Arkray, Japan). LT was defined as the exercise intensity not associated with an elevation in blood lactate concentration above baseline [23]. OBLA was defined as the exercise intensity identified by interpolation across the 4.0 mmol · L^−1^ lactate point [24].

During testing, respiratory gases were continuously measured with a metabolic cart (Metalyser 3B, Cortex, Leipzig, Germany) calibrated in accordance with the manufacturer’s instruction using Alpha gas standards. All participants maintained a respiratory exchange ratio (RER) < 0.85 at the first stage. All participants reached a stage where they achieved a failure to increase VO_2_ by 150 mL/min with increased workload and a RER ≥ 1.10.

VT2 was determined using the criteria of an increase in both the VE/VO_2_ and VE/VCO_2_ and a decrease in PETCO2 as previously described [25]. VO_2peak_ was determined as the highest 30 s oxygen uptake value recorded. The average treadmill speed over the final 60 s was recorded as peak 1 minute GET.

After a passive recovery of 15 minutes each runner performed a Maximal Anaerobic Running Test (MART) [26]. The MART consists of repeated 20-second sprints on a treadmill with 100 second passive rest periods. The speed of the first run was 14.5 km · h^−1^ and the inclination 5 degrees. The speed was increased by 1.5 km · h^−1^ every run until volitional exhaustion. vMART was calculated as the speed of the last completed 20-second run, plus 0.216 km · h^−1^ for each 2 seconds over 8 seconds of the subsequent uncompleted sprint. An uncompleted sprint of 8 seconds receives no adjustment, an uncompleted sprint of 10 seconds receives a 0.216 km · h^−1^ adjustment and 12 seconds receives a 0.432 km · h^−1^ adjustment.

### Outdoor running with Stryd

Each participant had a Stryd account set up for them by the researchers and access was shared between the researchers and participant. Participants were required wear Stryd for a minimum of 6 weeks for all their runs. This time frame was chosen from pilot testing on 5 individuals. While CP_STRYD_ is generated after only a few runs, it continues to update CP which becomes more stable with time. 6 weeks allowed for multiple estimates of CP for each participant. Laboratory testing procedures for each participant was also carried out within this same study period.

In addition to their usual running training, we referred participants to follow the advice given by Stryd for an accurate CP:

1. Long duration runs of 40–60 minutes at max effort or near max effort (time trial or 10k race) for those that are training for a half or full marathon distance.
2. Medium duration runs of 10–20 minutes at max effort or near max effort (time trial or 5k race)
3. A short max effort run that is 3–5 minutes in duration.
4. Short distance sprints or strides at max effort (10–30 seconds in duration).

To provide measures of running performance all participants were asked to compete at an outdoor 1500 m track race and / or a 5000 m track or flat road race. Participants were asked to race at their maximal effort and to race as close as possible to the period as they were running with Stryd. All events were official athletic track meets or on a measured and verified race courses. Race times were obtained from the individual event websites.

### Data analysis and statistics

For each subject, the final 10 s of power at every stage of GET and vMART was analysed for linearity using linear regression.

Following the outdoor running period with Stryd, all power data from each participants Stryd account was downloaded to Golden Cheetah (GC, version 3.5, open source, https://www.goldencheetah.org/). Golden Cheetah was used to obtain details of the number of runs, distance, training speed, duration, and elevation for each subject over this period of training. Golden Cheetah was also used to collate maximum mean power (MMP) for 3, 5, 10 and 20 minutes for each runner.

CP_STRYD_ from each participants Stryd account was collated. CP_STRYD_ is an auto-calculated CP from the Stryd app/website and uses an undisclosed power estimation algorithm. CP_STRYD_ was compared with MMP durations and with for Stryd power at ventilatory threshold 1 (VT1), LT, OBLA, VT2, VO_2peak_ and vMART. CP_STRYD_ was also examined as a predictor of VT1, LT, OBLA, VT2, VO_2peak_ and vMART using linear regression.

The four MMP durations (3, 5, 10 and 20 minutes) were used to calculate a CP for each subject (CP_CALC_). CP_CALC_ was calculated using the following linear power-time^−1^ model.

$$P=(W\prime /t)+CP_{CALC}$$

CP_CALC_ was then compared with CP_STRYD_ using Deming regression and a Bland-Altman plot to test for systematic bias. A two-one-sided t-tests (TOST) procedure was used to examine equivalence between CP_STRYD_ and CP_CALC_. As day to day variation of individual CP measures of ~5% have been previously reported [27], 5% was chosen as a priori acceptable limit to determine equivalence.

Stryd power at VT2 from laboratory testing was examined as predictor of CP_STRYD_ using both linear and non-linear regression regression and a Bland-Altman plot was used to test for systematic bias.

The two outdoor runs, 1-minute peak speed from the GET and vMART were used as performance measures. Linear regression was used to examine if CP_STRYD_ or Stryd data at 12.0 km · h^−1^ could predict these four measures of performance. This was also compared with laboratory results to predict performance.

Distributions were tested for normality with descriptive statistics and the D’Agostino and Pearson omnibus normality test to examine suitability for parametric testing. Comparisons were made with the paired t test.

All regressions were examined with the runs test and graphs of the residuals.

All data were analysed using Prism version 4.0 (GraphPad Version 4.01, San Diego, CA, USA, www.graphpad.com).

## RESULTS

20 participants completed laboratory testing procedures (Table 1). For all runners, linearity of Stryd power was excellent (R^2^ > 0.99) at 1% for GET stage and 5% incline for MART stage (R^2^ > 0.99).

**TABLE 1:** Participants and laboratory testing results.

	Age years	Weight Kg	Body Fat %	VT1 Km · h^−1^	LT Km · h^−1^	OBLA Km · h^−1^	VT2 Km · h^−1^	Peak GET Km · h^−1^	VO_2peak_	vMART Km · h^−1^
All (n = 20)										
Mean	39.5	71.4	14.3	11.9	12.6	14.2	14.7	17.0	3.93	21.8
SD	14.6	9.9	6.7	2.2	2.4	2.8	3.0	3.2	0.73	3.4

Male (n = 16)										
Mean	40.2	73.6	13.3	12.3	12.7	14.4	15.2	17.7	4.10	22.4
SD	14.7	9.5	6.7	2.1	2.4	2.9	2.8	3.1	0.70	3.3

Female (n = 4)										
Mean	34	63.0	22.7	10.0	11.1	12.0	12.3	14.6	3.20	19.2
SD	11.7	7.1	6.1	2.4	2.5	2.5	3.0	2.9	0.37	2.8

Note: Participant characteristics and laboratory graded exercise testing results are presented. VT1 = first ventilatory threshold, LT = lactate threshold, OBLA = onset of blood lactate accumulation, VT2 = second ventilatory threshold, Peak GET = peak 1 minute from graded exercise test, VO_2_ = maximum oxygen consumption, vMART = velocity maximal anaerobic running test, SD = standard deviation

The oxygen cost of running (2.92 ± 0.35 VO_2_ L min^−1^) at 12.0 km · h^−1^ had no relationship with Stryd economy measures form power (76 ± 11 W) nor form power ratio (0.30 ± 0.03). Also, there was no relationship between oxygen cost and vertical oscillation (8.7 ± 1.5 cm), cadence (84 ± 5 steps min^−1^), leg spring stiffness (11.5 ± 2.3 kN m-^1^) or GCT (235 ± 22 ms) at this same velocity.

Within 6 weeks of laboratory testing runners completed 54 ± 46 outdoor runs with Stryd, covering a distance of 464 ± 423 km in 39 ± 28 hours with an elevation gain of 4862 ± 3376 m. Training speed with Stryd was 11.9 ± 1.9 km · h^−1^. Stryd data from regular training was compared with Stryd data from laboratory testing which revealed OBLA and VT2 were the closest physiological thresholds to CP_STRYD_ (Table 2). CP_STRYD_ was not different (p > 0.05) from peak 20-minute power, power at OBLA or power at VT2. While speed at OBLA was slower (p < 0.01) compared with VT2 (Table 1), power at OBLA was not different (p = 0.07) compared with power at VT2 (Table 2).

**TABLE 2:** Stryd power from outdoor running compared with laboratory testing.

	Stryd Power (W) for runners during outdoor running					Stryd Power (W) for runners during laboratory testing					

	CP	3 min MMP	5 min MMP	10 min MMP	20 min MMP	VT1	LT	OBLA	VT2	Peak GET	MART
Min.	207	243	235	228	191	117	118	150	151	213	388
Mean	302	365	353	327	301	248	262	296	304	354	541
SD	58	78	74	65	69	51	46	56	65	66	108
Max.	393	512	487	422	406	320	325	369	393	449	703

**FIG. 1:**
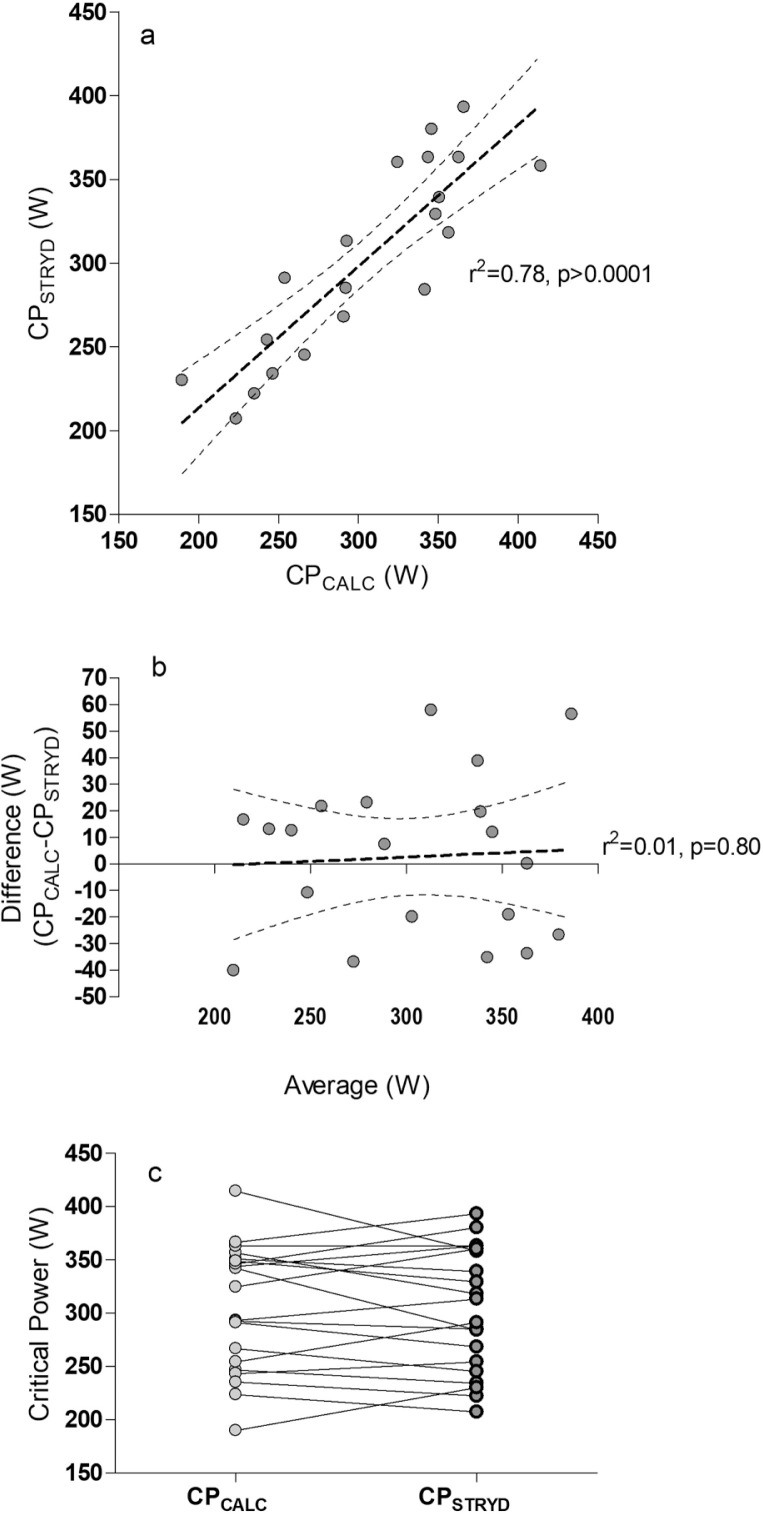
1a CPSTRYD is compared with vs CPCALC, Deming’s Regression Results: Slope = 0.97 ± 0.13, Y-intercept = 7.48 ± 39.98, SD of the residuals = 30.18, p<0.01. 1b There was no bias evident on the Bland Altman plot. 1c The two measures of CP appeared equivalent. Runs test p value = 0.77.

Stryd power data for 20 runners completing 54 ± 46 runs, covering a distance of 464 ± 423 km with Stryd are compared with Stryd power from laboratory testing. CP = critical power, MMP = maximum mean power, SD = standard deviation, VT1 = first ventilatory threshold, LT = lactate threshold, OBLA = onset of blood lactate accumulation, VT2 = second ventilatory threshold, Peak GET = peak 1 minute from graded exercise test., MART = maximal anaerobic running test,

CP_STRYD_ 302 ± 58 W was not different (p = 0.34) compared with CP_CALC_ 305 ± 60 W (Figure 1a). No bias was evident (Figure 1b). The TOST procedure (CP_CALC_ – CP_STRYD_, p = 0.34, Cohen’s d = 0.09 and CP_STRYD_ – CP_CALC_, p = 0.66, Cohen’s d = 0.09) confirmed that the two critical power estimates can be considered statistically equivalent (Figure 1c). The % differences between the two measures followed a normal distribution (0.8 ± 9.9%), ranged between -19.1 to 18.5%, 25^th^ and 75^th^ percentiles were -6.8 and 7.9%.

**FIG. 2:**
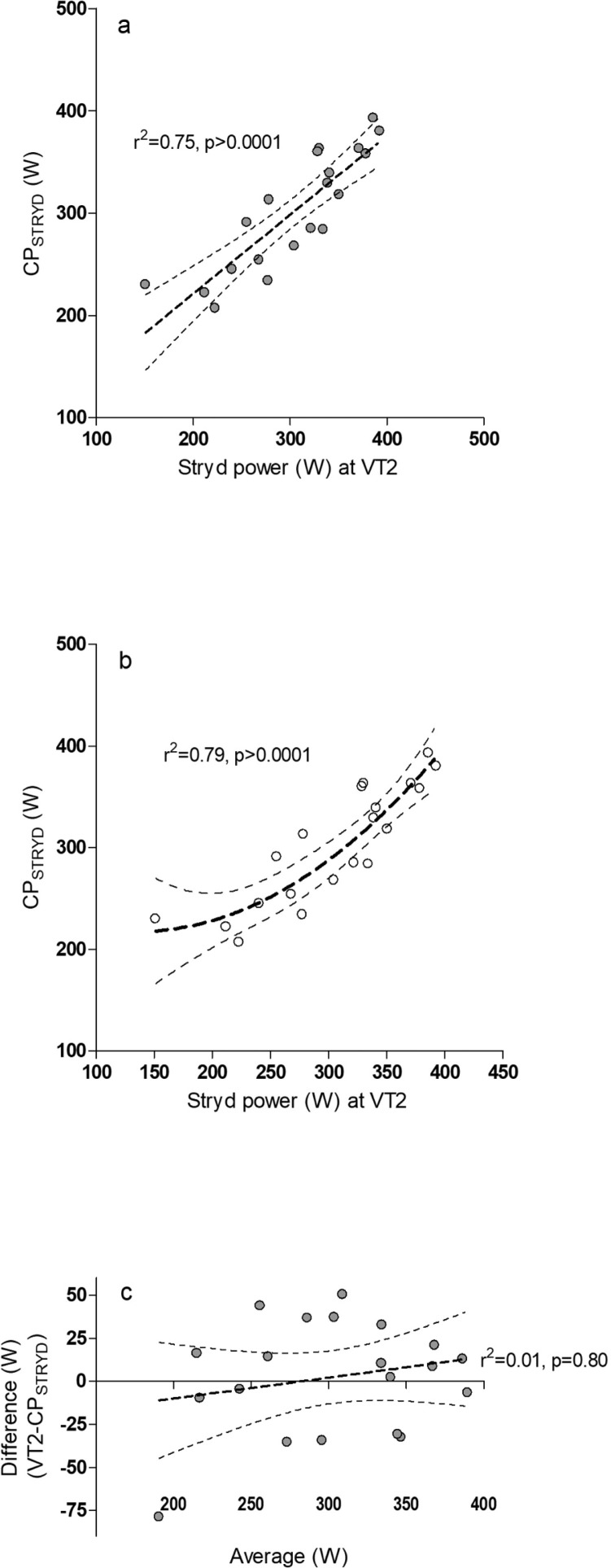
Stryd CP is compared with power at VT2 with (2a) Linear regression (Best-fit values: Slope 0.77 ± 0.11, Y-intercept when X=0.0 66.70 ± 32.89, X-intercept when Y=0.-86.35) and (2b) non-linear regression (Polynomial: Second Order (Y=A + B*X + C*X^2), Best-fit values A = 265.2, B = -0.70, C = 0.00) are compared. 2c A Bland Altman plot was used to examine bias and individual athlete differences. Runs test p value = 0.87.

As VT2 had a stronger relationship with CP_STRYD_ (R^2^ = 0.75) compared with OBLA (R^2^ = 0.72), VT2 was chosen as the threshold to directly compare with CP_STRYD_. While there was little difference between CP_STRYD_ and Stryd power at VT2 (figure 2a), the relationship may be best described as non-linear (2b) and individual differences of 20–40 W between CP_STRYD_ and power at VT2 were observed (Figure 2c).

13 participants ran a maximal 1500 m effort (320 ± 62 s) on a track or flat sealed surface within 33 ± 44 days of laboratory testing. 13 participants ran a maximal 5000 m effort (1174 ± 223 s) on a track or flat sealed surface within 34 ± 39 days of laboratory testing. 11 participants performed both distances. 7 participants could not compete at events because of COVID related event cancellations. These two outdoor runs and 1-minute peak speed from the GET and vMART were used as performance measures to compare Stryd data with laboratory tests (table 3).

**TABLE 3:** Comparing laboratory, Stryd and outdoor running as predictors of running performance.

Performance Measure	Best laboratory predictor	Best Stryd Predictor	Stryd GCT at 12 km hr^-1^	Running economy at 12 km h^-1^
5000 m time (s)	OBLA (Km · h−1)	CP (W/Kg)	NS	NS
	R^2^ = 0.95	R^2^ = 0.89		
	p < 0.01	p < 0.01		
	(n = 13)	(n = 13)		
	Runs p = 0.76	Runs p = 0.87		

1500 m time (s)	VT2 (Km · h−1)	CP (W/Kg)	GCT (ms)	NS
	R^2^ = 0.91	R^2^ = 0.86	R^2^ = 0.52	
	p < 0.01	p < 0.01	P < 0.01	
	(n = 13)	(n = 13)	(n = 13)	
	Runs p = 0.97	Runs p = 0.52	Runs p = 0.26	

1 minute peak speed during GET (Km · h−1)	VT2 (Km · h−1)	CP (W/Kg)	GCT (ms)	NS
	R^2^ = 0.94	R^2^ = 0.973	R^2^ = 0.27	
	p < 0.01	p < 0.01	p = 0.03	
	(n = 20)	(n = 20)	(n = 20)	
	Runs p = 0.07	Runs p = 0.43	Runs p = 0.55	

vMART speed (Km · h−1)	OBLA	20s PP (W)		
	R^2^ = 0.84	R^2^ = 0.88		
	p = 0.01	p < 0.01	NS	
	(n = 20)	(n = 20)		NS
	Runs p = 0.95	Runs p = 0.68		

The best laboratory-based data predictor of running performance is compared with the best Stryd data predictor for four performance measures. Stryd GCT is compared with running economy as a predictor. Each regression was examined with graphs of the residuals and the runs test. Runs test p values are included. PP = Peak Power. OBLA = onset of blood lactate accumulation, CP = critical power, VT2 = second ventilatory threshold,

While the two international class runners in our cohort had the lowest submaximal VO_2_ at 12.0 km · h^−1^, the oxygen cost of running was not predictive of performance. In contrast, Stryd GCT at 12.0 km · h^−1^ had an inverse relationship with performance and was predictive of 1-minute peak speed during GET, 1500 m time and was close to predicting (p = 0.07) 5000 m time. Further analysis revealed Stryd GCT at 12.0 km · h^−1^ also had inverse relationships with OBLA (r = -0.60. p = 0.04) and was close to significance with VO_2peak_ (r = -0.51. p = 0.05).

## DISCUSSION

We found that CP_STRYD_ generated from regular training over a six week period was equivalent to that calculated using an established CP model. However, individual runner variance between CP_STRYD_ and other CP models must be a consideration for runners and coaches. CP_STRYD_ was most similar in intensity to VT2 and OBLA and was predictive of outdoor and laboratory running performance. However, laboratory-based data were superior predictors of running performance when compared with CP_STRYD_. In addition, we found that Stryd running economy measures, form power and form power ratio, had little relationship with the oxygen cost of running. In contrast, GCT was inversely associated with performance when comparing runners at the same submaximal speed.

We found CP_STRYD_ (302 ± 58 W) was not different from CP_CALC_ (305 ± 60 W) estimated from a linear model using four MMP durations. In addition, we detected little bias between the two CP estimates. Day to day CP variation of ~5% has been previously reported for individual athletes [27]. As the differences between CP_STRYD_ and CP_CALC_ from our TOST procedure was not significant and is less (Cohen’s d = 0.09) than 5% we suggest that the two CP estimates can be considered equivalent. It must be noted that some participants did record differences between the two CP estimates. While physiologically, CP supposedly represents a single boundary for an individual athlete between steady-state and non-steady state exercise intensity [12], different protocols and models are known to affect its determination [15]. CP_STRYD_ is an unknown unknown proprietary algorithm and the power time durations used in its estimation are not published for commercial reasons. Individual runner variance between the two CP estimates is somewhat an expected finding. Coaches and runners should be aware that any method of determining CP may not be interchangeable with other methods. However, CP_STRYD_ appears to be an acceptable method of determining CP in running when compared with previously published linear models.

CP_STRYD_ intensity closely resembles VT2 and OBLA which are thresholds frequently used as guides for training intensity distribution or “polarised training” in endurance athletes [28, 29]. CP_STRYD_ would thus appear to facilitate polarised training at recommend intensity distributions, without the need for laboratory testing. There may be limitations for using CP_STRYD_ as a threshold. Our results suggest for participants with lower CP_STRYD_ values (~220 W), VT2 may occur at a lower intensity compared with CP_STRYD_. Also, individual subject differences of 20–40 W (7–13%) between CP_STRYD_ and power at VT2 were observed. Although CP_STRYD_ intensity may be similar to VT2, we cannot discount that it may be an independent physiological threshold. Also, it should be noted that we did not determine MLSS which has been suggested as a true representation of the boundary between steady-state and non-steady state exercise intensity [17, 18, 30]. In spite of these limitations, we speculate that individuals who experience an increase in CP_STRYD_ (W/kg) would also likely improve endurance performance. Although we did not specifically compare changes in CP_STRYD_ with changes in performance, some runners in our cohort did experience an increase in CP_STRYD_ over the training period that appeared to coincide with an increase in performance. Further research is required to confirm if changes in CP_STRYD_ with correlate with changes in performance.

We found no difference (p = 0.07) in Stryd power between OBLA and VT2 (296 vs 304 W), yet running speed at OBLA was slower (p < 0.01) than VT2 (14.2 vs 14.7 km h^−1^). Similar OBLA and VT2 velocity differences have been reported previously in runners [25]. The fact that velocity and not power was different, might suggest that velocity is a more precise measure of intensity compared with Stryd power. However, even a 1% change in grade substantially changes the energetic cost of running [22], while Stryd power, unlike velocity, accounts for both elevation and wind. We found Stryd power linearity for flat and incline treadmill running was excellent (R^2^ > 0.99). Thus, we speculate that Stryd power is likely to be superior compared with velocity for any individual runner seeking to measure running intensity where a change in grade result in changes in velocity.

CP_STRYD_ (W/Kg) was able to explain 73–89% of the variance in running performance for 1500 m, 5000 m and GET peak speed. However, for all three of these measures of running performance, laboratory testing was superior to Stryd CP and explained 91–95% of the variance. Treadmill velocities during GET were the most predictive of performance which is similar to previous research [31]. While the two international class runners in our cohort had lowest oxygen cost at submaximal speed, this was not predictive of any performance in this study. In contrast, Stryd GCT had significant inverse relationships with peak GET speed, VO_2peak_, OBLA, and outdoor running performance. Lower GCT has been associated with greater economical running in elite runners [32]. While laboratory exercise testing remains the gold standard to predict endurance performance, CP_STRYD_ is highly predictive of performance and Stryd GCT may also be a useful field-based parameter.

A major limitation from our study was that COVID restrictions prevented a single time trial. Thus, for our outdoor trials, participants encountered varying running conditions that we are unable to control for such as different weather, temperature and courses. Nevertheless, even with this limitation, CP_STRYD_ was highly predictive of outdoor performance. It must also be noted that our runners could not have feasibility raced one another in any meaningful way. Even a 1500 m track based time trail would result in some participants being lapped more than once. We believe this heterogeneity of our population can also be considered a strength as runners are not a homogenous group and running power will appeal to many runners of different abilities. Predictive measures such as VO_2peak_ are more predictive of performance in heterogenous populations [33]. A homogenous group such as elite runners would be likely to generate weaker relationships with Stryd than we have found. This also warrants further research. One possible limitation is that we did not determine MLSS and this also would be one area for further research. Also, a possible limitation is that while CP_STRYD_ was statistically equivalent to CP_CALC_, some participants recorded differences between the two estimates. Coaches and runners need to remain aware that CP estimates are highly influenced by both trial protocols and the mathematical models employed [15]. Even when using the same protocol and model a ~5% day to day variance should be expected [27].

## CONCLUSIONS

In conclusion, CP_STRYD_ generated from outdoor running is equivalent to that calculated using an established CP model. CP_STRYD_ appears to most similar in intensity to VT2 or OBLA and may offer a useful guide for training intensity distribution or polarised training. CP_STRYD_ is highly predictive of running performance although laboratory-based data are superior predictors. While Stryd was not predictive of the oxygen cost of running when comparing runners at the same submaximal speed, Stryd GCT is inversely associated with performance and may be a useful field-based parameter. Stryd offers meaningful data for runners including a realistic estimate of CP.

## References

[1] Jaén-Carrillo D, Roche-Seruendo LE, Cartón-Llorente A, Ramírez-Campillo R, García-Pinillos F. Mechanical power in endurance running: a scoping review on sensors for power output estimation during running. Sensors. 2020; 20(22):6482. 33202809 10.3390/s20226482 PMC7696724

[2] García-Pinillos F, Latorre-Román PÁ, Roche-Seruendo LE, García-Ramos A. Prediction of power output at different running velocities through the two-point method with the Stryd™ power meter. Gait Posture. 2019; 68:238–43. 30528962 10.1016/j.gaitpost.2018.11.037

[3] Cerezuela-Espejo V, Hernández-Belmonte A, Courel-Ibáñez J, Conesa-Ros E, Mora-Rodríguez R, Pallarés JG. Are we ready to measure running power? Repeatability and concurrent validity of five commercial technologies. Eur J Sport Sci. 2021; 21(3):341–50. 32212955 10.1080/17461391.2020.1748117

[4] Austin CL, Hokanson JF, McGinnis PM, Patrick S. The relationship between running power and running economy in well-trained distance runners. Sports. 2018; 6(4):142. 30404176 10.3390/sports6040142 PMC6317050

[5] García-Pinillos F, Latorre-Román PÁ, Soto-Hermoso VM, Párraga-Montilla JA, Pantoja-Vallejo A, Ramírez-Campillo R, Roche-Seruendo LE. Agreement between the spatiotemporal gait parameters from two different wearable devices and high-speed video analysis. PLoS One. 2019; 14(9):e0222872. 31550296 10.1371/journal.pone.0222872 PMC6759187

[6] Navalta JW, Montes J, Bodell NG, Aguilar CD, Radzak K, Manning JW, DeBeliso M. Reliability of trail walking and running tasks using the Stryd power meter. Int J Sports Med. 2019; 40(08):498–502. 31288288 10.1055/a-0875-4068

[7] Pinedo-Jauregi A, Garcia-Tabar I, Carrier B, Navalta JW, Cámara J. Reliability and validity of the Stryd Power Meter during different walking conditions. Gait Posture. 2022; 92:277–83. 34896839 10.1016/j.gaitpost.2021.11.041

[8] García-Pinillos F, Soto-Hermoso VM, Latorre-Román PÁ, Párraga-Montilla JA, Roche-Seruendo LE. How Does Power During Running Change when Measured at Different Time Intervals? Int J Sports Med. 2019; 40(09):609–13. 31295744 10.1055/a-0946-2159

[9] Imbach F, Candau R, Chailan R, Perrey S. Validity of the Stryd power meter in measuring running parameters at submaximal speeds. Sports. 2020; 8(7):103. 32698464 10.3390/sports8070103 PMC7404478

[10] García-Pinillos F, Roche-Seruendo LE, Marcén-Cinca N, Marco-Contreras LA, Latorre-Román PA. Absolute reliability and concurrent validity of the Stryd system for the assessment of running stride kinematics at different velocities. J Strength Cond Res. 2021; 35(1):78–84. 29781934 10.1519/JSC.0000000000002595

[11] Jones AM, Vanhatalo A, Burnley M, Morton RH, Poole DC. Critical power: implications for determination of VO_2_max and exercise tolerance. Med Sci Sports Exerc. 2010; 42(10):1876–90. 20195180 10.1249/MSS.0b013e3181d9cf7f

[12] Vanhatalo A, Jones AM, Burnley M. Application of critical power in sport. Int J Sports Physiol Perform. 2011; 6(1):128–36. 21487156 10.1123/ijspp.6.1.128

[13] Mitchell EA, Martin NR, Bailey SJ, Ferguson RA. Critical power is positively related to skeletal muscle capillarity and type I muscle fibers in endurance-trained individuals. J Appl Physiol. 2018; 125(3):737–45. 29878875 10.1152/japplphysiol.01126.2017

[14] Hall EC, Semenova EA, Bondareva EA, Borisov OV, Andryushchenko ON, Andryushchenko LB, Zmijewski P, Generozov EV, Ahmetov II. Association of muscle fiber composition with health and exercise-related traits in athletes and untrained subjects. Biol Sport. 2021; 38(4):659. 34937976 10.5114/biolsport.2021.102923 PMC8670815

[15] Maturana FM, Fontana FY, Pogliaghi S, Passfield L, Murias JM. Critical power: How different protocols and models affect its determination. J Sci Med Sport. 2018; 21(7):742–7. 29203319 10.1016/j.jsams.2017.11.015

[16] Galán-Rioja MÁ, González-Mohíno F, Poole DC, González-Ravé JM. Relative proximity of critical power and metabolic/ ventilatory thresholds: systematic review and meta-analysis. Sports Med. 2020; 50(10):1771–83. 32613479 10.1007/s40279-020-01314-8

[17] Gorostiaga EM, Sánchez-Medina L, Garcia-Tabar I. Over 55 years of critical power: Fact or artifact? Scan. J Med Sci Sports. 2022; 32(1):116–24. 10.1111/sms.14074 34618981

[18] Iannetta D, Ingram CP, Keir DA, Murias JM. Methodological Reconciliation of CP and MLSS and Their Agreement with the Maximal Metabolic Steady State. Med Sci Sports Exerc. 2021. 10.1249/MSS.0000000000002831 34816811

[19] Keir DA, Mattioni Maturana F, Iannetta D, Murias JM. Comment on:“Relative proximity of critical power and metabolic/ ventilatory thresholds: systematic review and meta-analysis”. Sports Med. 2021; 51(2):367–8. 33108653 10.1007/s40279-020-01365-x

[20] Nevill M, Garrett A, Maxwell N, Parsons K, Norwitz A. Thermal strain of intermittent and continuous exercise at 10 and 35 C in man. J Physiol. 1995; 486(1):124–5.

[21] Allen H, Coggan AR, McGregor S. Training and racing with a power meter Boulder: VeloPress; 2019.

[22] Jones AM, Doust JH. A 1% treadmill grade most accurately reflects the energetic cost of outdoor running. J Sports Sci. 1996; 14(4):321–7. 8887211 10.1080/02640419608727717

[23] Weltman A, Snead D, Stein P, Seip R, Schurrer R, Rutt R, Weltman J. Reliability and validity of a continuous incremental treadmill protocol for the determination of lactate threshold, fixed blood lactate concentrations, and VO_2_max. Int J Sports Med. 1990; 11(01):26–32. 2318561 10.1055/s-2007-1024757

[24] Sjödin B, Jacobs I. Onset of blood lactate accumulation and marathon running performance. Int J Sports Med. 1981; 2(01):23–6. 7333732 10.1055/s-2008-1034579

[25] Cerezuela-Espejo V, Courel-Ibáñez J, Morán-Navarro R, Martínez-Cava A, Pallarés JG. The relationship between lactate and ventilatory thresholds in runners: validity and reliability of exercise test performance parameters. Front Physiol. 2018; 9:1320. 30319439 10.3389/fphys.2018.01320 PMC6167480

[26] Rusko H, Nummela A, Mero A. A new method for the evaluation of anaerobic running power in athletes. Eur J Appl Physiol Occup Physiol. 1993; 66(2):97–101. 8472703 10.1007/BF01427048

[27] Poole DC, Burnley M, Vanhatalo A, Rossiter HB, Jones AM. Critical power: an important fatigue threshold in exercise physiology. Med Sci Sports Exerc. 2016; 48(11):2320. 27031742 10.1249/MSS.0000000000000939 PMC5070974

[28] Seiler KS, Kjerland GØ. Quantifying training intensity distribution in elite endurance athletes: is there evidence for an “optimal” distribution? Scand J Med Sci Sports. 2006; 16(1):49–56. 16430681 10.1111/j.1600-0838.2004.00418.x

[29] Seiler S. What is best practice for training intensity and duration distribution in endurance athletes? Int J Sports Physiol Perform. 2010; 5(3):276–91. 20861519 10.1123/ijspp.5.3.276

[30] Garcia-Tabar I, Gorostiaga EM. Comment on:“Relative Proximity of Critical Power and Metabolic/Ventilatory Thresholds: Systematic Review and Meta-Analysis”. Sports Med. 2021; 51(9):2011–3. 34165764 10.1007/s40279-021-01497-8

[31] Stratton E, O’brien B, Harvey J, Blitvich J, McNicol A, Janissen D, Paton C, Knez W. Treadmill velocity best predicts 5000-m run performance. Int J Sports Med. 2009; 30(01):40–5. 19202577 10.1055/s-2008-1038761

[32] Santos-Concejero J, Granados C, Irazusta J, Bidaurrazaga-Letona I, Zabala-Lili J, Tam N, Gil S. Differences in ground contact time explain the less efficient running economy in North African runners. Biol Sport. 2013; 30(3):181. 24744486 10.5604/20831862.1059170 PMC3944563

[33] Legaz-Arrese A, Munguía-Izquierdo D, Nuviala AN, Serveto-Galindo O, Urdiales DM, Masía JR. Average VO_2_max as a function of running performances on different distances. Sci Sports. 2007; 22(1):43–9.

